# The factors limiting the diagnostic accuracy of myocardial perfusion cardiac magnetic resonance imaging: coronary flow reserve and amount of myocardial scar

**DOI:** 10.1186/1532-429X-14-S1-O88

**Published:** 2012-02-01

**Authors:** Masaki Ishida, Shingo Kato, Nanaka Ishida, Motonori Nagata, Kakuya Kitagawa, Hiroshi Nakajima, Shiro Nakamori, Hajime Sakuma

**Affiliations:** 1 Radiology, Mie University Hospital, Tsu, Japan; 2 Cardiology, Mie University Hospital, Tsu, Japan

## Background

Perfusion cardiac magnetic resonance (CMR) imaging has emerged as an important clinical tool for the accurate detection of myocardial ischemia caused by significant coronary artery disease (CAD), whereas most previous studies demonstrated the diagnostic accuracy of perfusion CMR in a highly selected patient population such as those without previous myocardial infarction or lower ejection fraction (EF) etc. In the real clinical practice, however, we may encounter the patients with multiple factors affecting the accurate CMR myocardial perfusion assessment. The aim of this study was to identify the factors reducing the sensitivity of perfusion CMR for predicting significant CAD determined by the coronary angiography (CAG).

## Methods

181 patients with known or suspected CAD underwent both CMR imaging and CAG. Patients who had previously undergone coronary artery bypass graft were excluded. CMR imaging was performed at either 1.5T or 3T system. Perfusion CMR images were acquired at rest and during ATP stress with Gadolinium (Gd) dose of 0.05 (0.03) mmol/kg at 1.5T (3T). Coronary sinus flow measurement was performed at rest and during stress using phase contrast cine MR imaging to calculate coronary flow reserve (CFR). Short axis cine stack was obtained to calculate LV volume and function. Late gadolinium enhancement (LGE) imaging was performed using cumulative Gd dose of 0.15mmol/kg. Presence or absence of ischemia in individual coronary territories was visually determined on perfusion CMR images. Percentage myocardium affected by scar in the territories (%LGE) was then calculated. Coronary artery stenosis ≥70% was considered significant. Candidate variables to be included in multivariate analysis were: LV end-diastolic volume, cardiac output, EF, CFR and %LGE.

## Results

Of 543 coronary territories, 266 corresponded to significant CAD. Of these, 61 (22.9%) were judged as absence of ischemia (false negative). Per-territory analysis demonstrated the sensitivity of perfusion CMR for predicting significant CAD was 77.1% whilst overall per-patient analysis indicated a sensitivity of 85.5%. In multiple logistic regression analysis, the independent factors to have a significant relationship with the false negative were CFR (Odds ratio, 0.59; 95% confidence interval (CI), 0.44-0.80) and %LGE (1.03; 1.02-1.04) (table 1). Using a threshold of CFR=2 and %LGE=25%, smaller CFR and larger %LGE groups had substantially lower sensitivity (table 2).

**Table 1:** Results of the multiple logistic regression analysis

	Significance probability (p)	Odds ratio	95% CI	
			
			lower	upper
CFR	0.001	0.591	0.439	0.795

%LGE	0.001	1.025	1.011	1.04

**Table 2:** Diagnostic performance of perfusion CMR imaging

			n	Sensitivity	p*	PPV	NPV	Specificity
Per Patient Analysis			181	85.5	-	95.4	58.8	83.3

	all		543	77.1	-	89.1	80.5	91.0
	
	CFR	≦2	153	70.8	0.056	91.3	58.9	86.0
				
Per Territory Analysis		2<	390	81.0		88.0	87.1	92.1
	
	%LGE	≦25%	484	80.5	0.001	89.4	84.6	91.8
				
		25%<	59	56.1		88.5	45.5	83.3

*: Chi-square test

## Conclusions

The current results indicate that reduced CFR and increased myocardial scar are associated with reduced sensitivity in detecting myocardial ischemia on stress CMR perfusion images.

## Funding

Nothing to disclose.

